# Assessment of Pitfalls in an AI-Based Pill-Counting Application

**DOI:** 10.7759/cureus.90837

**Published:** 2025-08-23

**Authors:** Namie Tanamachi, Takahiro Amemiya, Takashi Tomita

**Affiliations:** 1 Department of Pharmacy, International University of Health and Welfare Mita Hospital, Minato, JPN; 2 Department of Pharmaceutical Sciences, Teikyo Heisei University, Nakano, JPN; 3 Department of Pharmaceutical Sciences, School of Pharmacy, International University of Health and Welfare, Narita, JPN

**Keywords:** ai, medical safety, pharmacist, pill-counting application, yakushimarukazuko

## Abstract

Artificial intelligence (AI)-based pill-counting applications have been introduced into clinical practice to streamline the work of pharmacists. However, no study has evaluated the safety of these drugs. The purpose of this study was to evaluate AI's ability to recognize Yakushimarukazuko^®^ (NeoX Inc., Tokyo, Japan) and identify operational issues. The results confirmed that the transparency, overlap, and direction of the supplements used in the assessment were important factors in AI-based recognition. Accurately identifying and addressing such operational issues with AI-based pill-counting applications will contribute to minimizing the risk of errors and, in turn, enhance medical safety.

## Introduction

Dispensing errors constitute a clinical problem, as they frequently occur in healthcare settings [1]. Medication errors are the leading cause of medical errors that can harm patients and cause adverse drug events [2,3]. Therefore, although pharmacists take measures to prevent dispensing errors, they cannot completely prevent them [4].

In recent years, artificial intelligence (AI) has been incorporated into healthcare to ensure patient safety by improving error detection, patient stratification, and medication management by healthcare professionals [5]. AI image recognition is a technology that uses machine learning techniques such as deep learning to recognize and classify objects and patterns in images [6]. Computerized decision support systems have been evaluated as effective in avoiding prescription errors, such as inappropriate drug selection, drug interactions, and medication errors [7]. AI algorithms also analyze patient data to identify potential drug interactions, optimize dosing, and predict adverse reactions [8].

Yakushimarukazuko^®^ (NeoX Inc., Tokyo, Japan) is a free application designed to streamline the work of pharmacists with the ability to instantly count individual tablets using AI technology [9]. This application eliminates the need for pharmacists to visually count drugs, leading to a significant reduction in work time. However, image recognition using AI is imperfect. In Japan, pharmacists bear the ultimate responsibility for dispensing errors, including those resulting from system errors. Therefore, pharmacists must be familiar with AI systems and possess the knowledge and skills to operate them properly [8]. This study aimed to understand the pitfalls of using AI-based pill-counting applications for safe use.

## Technical report

Methods

Materials

Three different supplements were used to evaluate the usefulness of the tablet-counting application. Samples 1 and 2 were procured from iHerb, LLC., USA (lot: 3337193; details unknown). Sample 3 was procured from Elumild, Inc. (Osaka, Japan; lot: +EA1). Sample 1 represents a capsule dosage form, Sample 2 represents a permeable dosage form, and Sample 3 represents a tablet dosage form.

AI-Based Pill Counting Application

**Figure 1:**
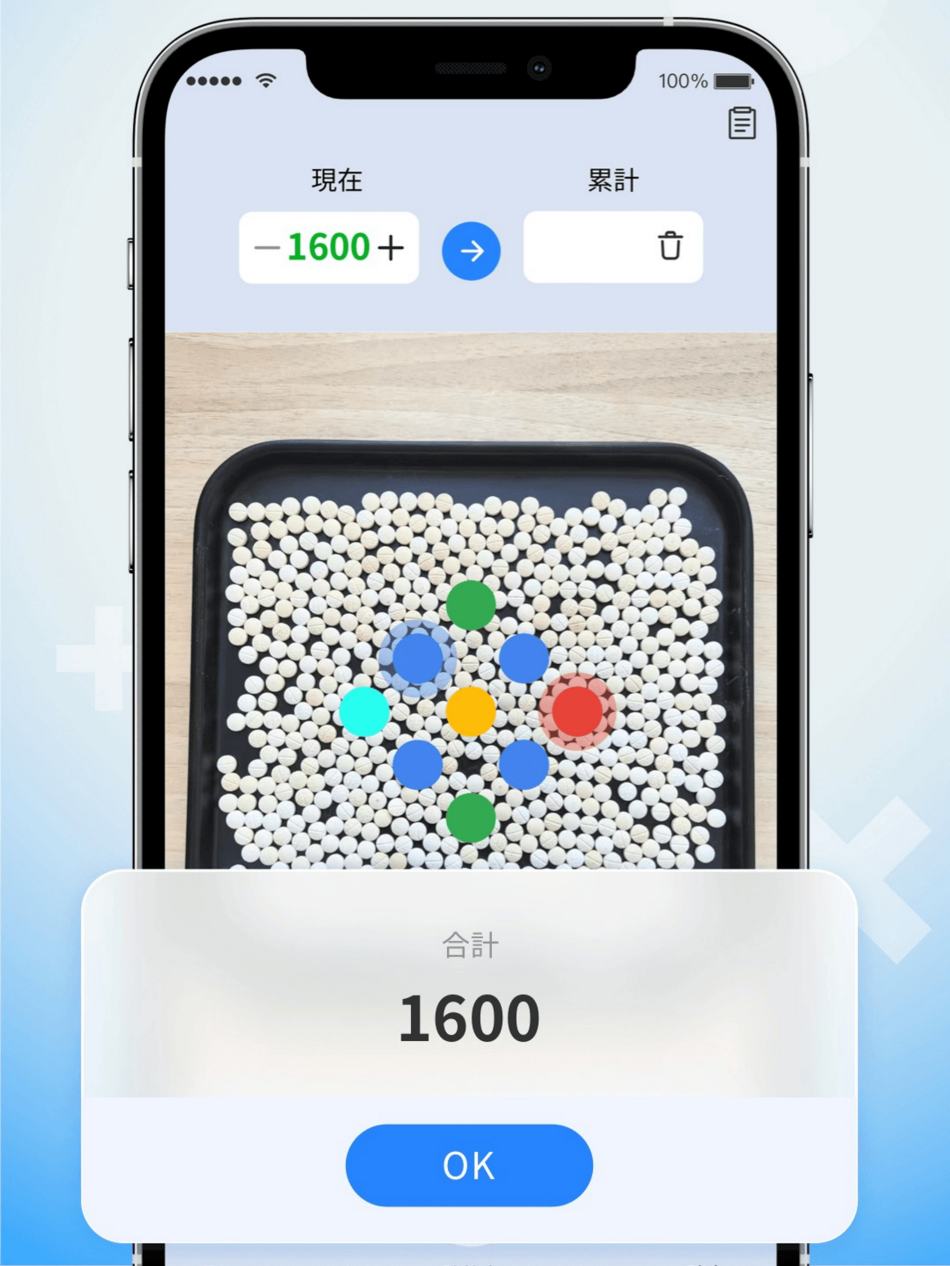
Overview of Yakushimarukazuko® [10]

Yakushimarukazuko^®^ was used as the AI-based pill-counting application (Figure 1) [10]. This application uses high-speed AI to quickly count the number of pills in pictures taken with a mobile phone. The supplements were placed on the tray, and pictures were taken using a mobile phone with a downloaded application. Simultaneously with photography, AI technology analyzed the images, and the number of supplements was instantly measured.

Verification of Safety in the AI-Based Pill Counting Application

This study evaluated whether changing the way supplements were placed on the trays would pose a problem for the AI technology that determines the number of pills.

Results

Assessment of Pitfalls in the AI-Based Pill Counting Application

**Figure 2:**
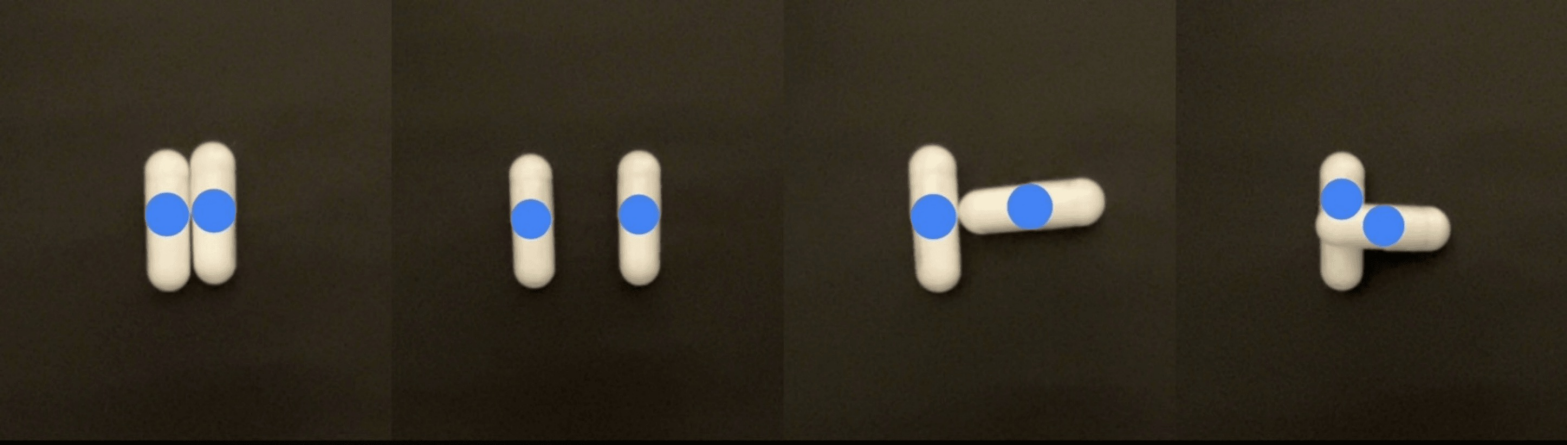
AI recognition status in Sample 1 image AI: artificial intelligence

To measure the tablets on a tray using AI, the tablets must be placed in various orientations and measured uniformly. Therefore, supplements placed in various orientations were measured using an AI-based pill-counting application to evaluate their safety profile. The AI application correctly recognized the photograph of Sample 1 (Figure 2).

**Figure 3:**
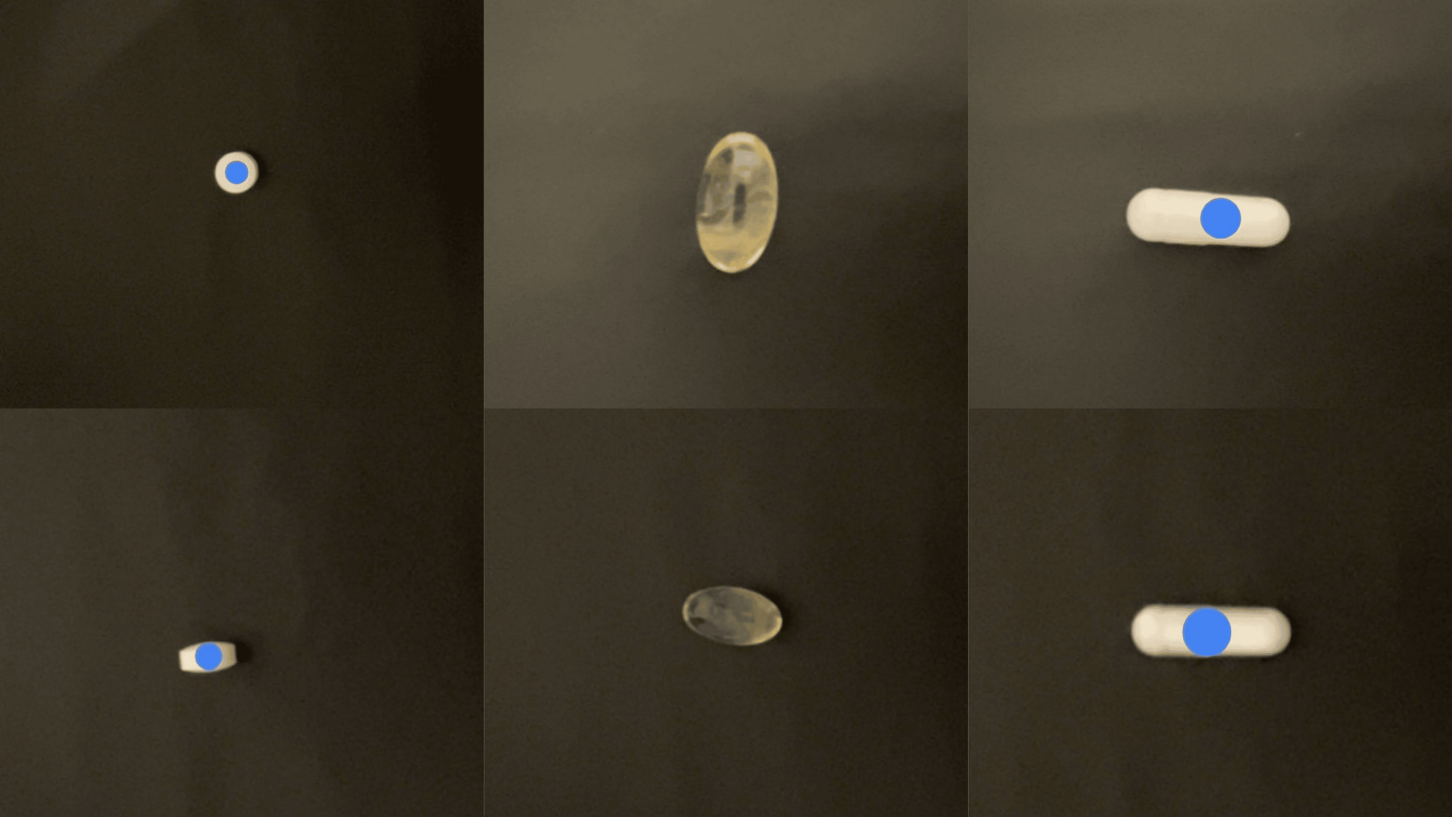
AI recognition status in images of Samples 1, 2, and 3 single agents AI: artificial intelligence

However, an AI recognition error was identified in the photographic image of Sample 2 (Figure 3).

**Figure 4:**
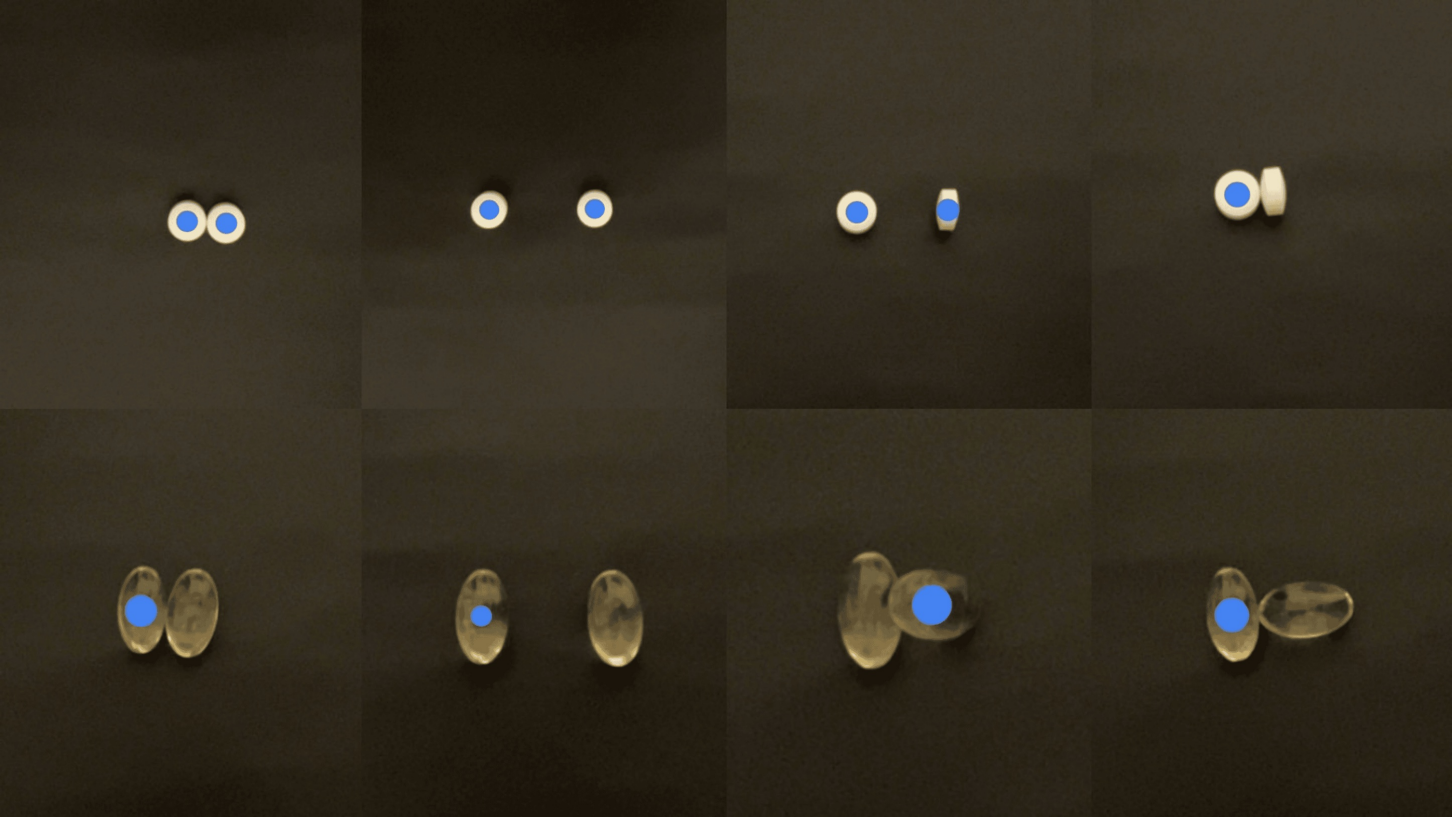
AI recognition status of images when Samples 2 and 3 are placed at various distances and directions

Furthermore, when two of the samples in Sample 3 were placed close together in a vertical orientation, the AI application failed to recognize them (Figure 4).

**Figure 5:**
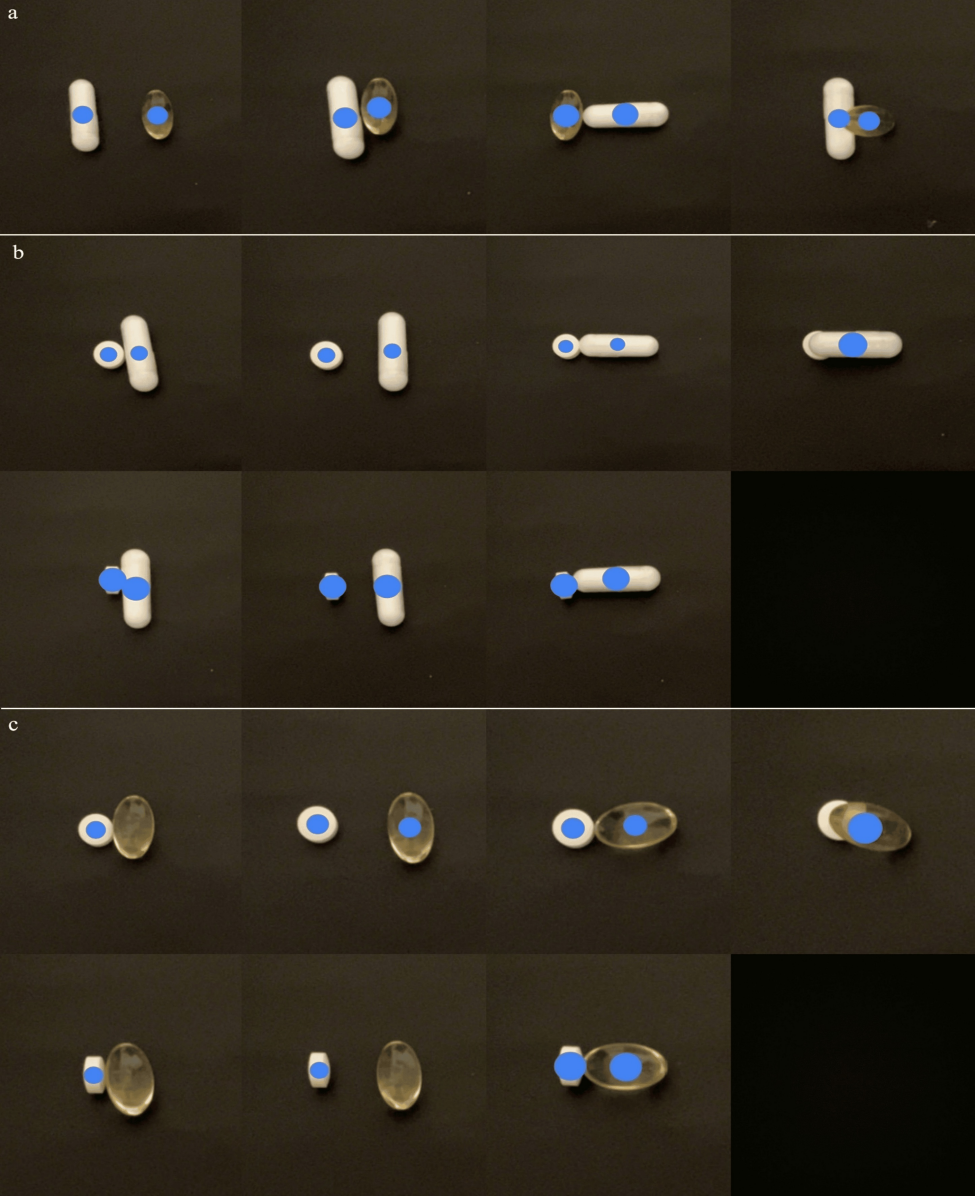
AI recognition status of images when supplements were superimposed on each other (a) AI recognition status of images when Samples 1 and 2 are placed at various distances and directions. (b) AI recognition status of images when Samples 1 and 3 are placed at various distances and directions. (c) AI recognition status of images when Samples 2 and 3 are placed at various distances and directions. AI: artificial intelligence

We then evaluated the recognition of the supplements by the AI when they were superimposed on each other. The AI recognized the combination of supplements 1 and 2 without problems, but failed to correctly recognize supplements 1 and 3 or 2 and 3 (Figure 5a-5c).

## Discussion

In this study, we identified, for the first time, that transparency, overlap, and direction of supplements are important factors in AI recognition as pitfalls in the AI-based pill-counting application Yakushimarukazuko^®^. This application is expected to prevent pharmacists from making visual drug-counting errors and significantly reduce their workload. Furthermore, this system requires no communication and can be used anytime, anywhere, and free of charge [11]. In addition, it may be useful for measuring individual drugs filled in cassettes used in single-packet dispensing machines and drugs brought in during hospitalization. The benefit of reducing the time spent on these tasks is that more time can be allocated to other tasks. The application also helps reduce the burden of ordering by accurately identifying the number of tablets remaining and managing their quantities.

Several challenges are associated with the use of AI-based pill-counting applications. This study revealed that transparency, overlap, and direction of supplements are important factors in AI recognition. The detailed cause is unknown; however, the need to learn using AI has been highlighted [12]. AI must learn about image recognition when overlapping with other drugs, in addition to orientation differences in the same drug. Learning about new drugs is also important. Therefore, to reduce the frequency of false detections, it is necessary to evaluate detection accuracy in advance using the drugs that will be used at each facility.

A limitation of this study is that only three types of supplements were evaluated. The ability of AI to recognize pharmaceuticals has not yet been evaluated; therefore, further verification is necessary. In addition, no comparisons were made using different lots of the supplements used in this study, and lot information for Sample 2 was unknown.

## Conclusions

AI-based pill-counting applications are essential for streamlining the workflows of pharmacists. However, implementing these systems requires a multifaceted approach to overcome operational challenges, including not only the technical aspects of the system but also adequate training, user education, and continuous improvement measures. Minimizing the risk of errors by implementing and effectively operating appropriate systems is crucial. Accurately identifying and properly addressing the operational challenges that arise when using AI-based tablet-counting applications will minimize the risk of errors and improve medical safety.

## References

[1] Bohand X, Simon L, Perrier E, Mullot H, Lefeuvre L, Plotton C (2009). Frequency, types, and potential clinical significance of medication-dispensing errors. Clinics (Sao Paulo).

[2] Phillips DP, Christenfeld N, Glynn LM (1998). Increase in US medication-error deaths between 1983 and 1993. Lancet.

[3] Ferner RE, Aronson JK (2000). Medication errors, worse than a crime. Lancet.

[4] Ikeda K, Kominami S, Morita S, Kita H, Uno M (2011). Effectiveness of dedicated dispensing paper in preventing dispensing errors (Article in Japanese). Iryo Yakugaku.

[5] Oliva A, Altamura G, Nurchis MC (2022). Assessing the potentiality of algorithms and artificial intelligence adoption to disrupt patient primary care with a safer and faster medication management: a systematic review protocol. BMJ Open.

[6] Tian Y (2020). Artificial intelligence image recognition method based on convolutional neural network algorithm. IEEE Access.

[7] Damiani G, Altamura G, Zedda M (2023). Potentiality of algorithms and artificial intelligence adoption to improve medication management in primary care: a systematic review. BMJ Open.

[8] Takase T, Muroi N, Hashida T (2025). Use of robotics and AI to transform dispensing and drug therapy as well as shaping the future of pharmacy education in Japan. J Asian Assoc Sch Pharm.

[9] (2025). Yakushimarukazuko (Website in Japanese). https://www.yakumaru.ai/kazuko.

[10] (2025). Introduction of yakushimarukazuko application (Website in Japanese). https://play.google.com/store/apps/details?id=com.neox.countpill&pli=1.

[11] (2025). Explanation of the function of yakushimarukazuko (Website in Japanese). https://pub-mediabox-storage.rxweb-prd.com/exhibitor/products/exh-db67f0f9-d297-4ef4-bae4-e09556599db0/product-documents/pro-c1c7218c-97d0-430d-9ad2-c2114bb21826/191e3d94-3ecc-4a5e-892a-d6a63e0eed91.pdf.

[12] Hamamoto R (2022). Clinical applications of artificial intelligence (AI) technology: focusing on medical image analysis (Article in Japanese). JPN J Neurosurg (Tokyo).

